# Monthly Summary

**Published:** 1878-06

**Authors:** 


### MONTHLY SUMMARY.

---

*Gelseminum Sempervirens in Neuralgia.*—The action of this drug in affections of a neuralgic character, says the *Medical Examiner*, has recently been studied by Dr. Emery-Heroguelle, who made it the subject of his inaugural thesis. A summary of his observations appeared in a recent number of the *Paris Medical.* Taken in a large dose gelseminum produces frontal headache, stunning, visual troubles, diplopia, contraction of the pupil, and dropping of the upper eyelid. There is also weakness of the legs. The author reports six cases of intoxication from this drug, taken in mistake. Gelseminum is administered in powder or in pills in the dose of three-fourths of a grain to three grains of the powder of the roots. It may also be given in the form of tincture, made with 100 parts of alcohol at 60° to 5 parts of the powdered root. The dose is from 40 to 80 drops. A syrup may be also made by adding 50 parts of the tincture to

1000 of simple syrup. M. Dujardin-Beaumetz has also had prepared an aqueous extract and an alcoholic extract. M. Emery-Heroguelle reports thirty-one observations collected in the service of M. Dujardin-Beaumetz, and from foreign journals, all of which refer to the action of the drug on neuralgia. From an analysis of the results, it appears that gelseminum may be especially looked upon as an anti-neuralgic; that it acts favorably in cases of dental neuralgia of the fifth pair, of the frontal, temporal, supra-, and infra-orbital nerves, the brachial plexus, the intercostal and ilio-lumbar nerves. Sciatic neuralgia appears to resist, rather more than other neuralgias, the calming effects of this tincture. Dr. Ortille, of Lille, however, succeeded in curing with this remedy a patient who had suffered for a long time from sciatica which resisted all sorts of therapeutic means. The author considers gelseminum to be a powerful sedative in neuralgia, especially in those varieties which are not accompanied by that local fluxion in the affected point. Favorable results have also been seen in hemicrania.—*Med. and Surg. Reporter.*

---

*The Effects of Lymph on Cicatrization.*—Professor Koeberle, of Strasburg, states that lesions or cuts intersecting the lymphatics are more difficult than others to heal by the first intention. Moreover, the lymph, easily becoming puriform, gives rise to the sanies which, infiltrating the tissues, occasions erysipelas, secondary hemorrhages, and other accidents consecutive upon a wound. This stagnation of the sanies is rendered evident by redness and pain occurring at some point in the line of the cicatrix. It then becomes necessary to allow of its escape by opening the wound at this point. Observation shows that in the middle line of the body, where there are few lymphatics, immediate union often occurs: and further, that longitudinal incisions, which do less injury to the lymphatics, heal better than horizontal ones. Professor Koeberle hence draws the conclusion that, when practicable, incisions should be made paralled to, and not across, lymphatic vessels.—*Med. and Surg. Reporter.*

---

*Absurd.*—The following under the title of "Source of Artificial Teeth," is going the rounds of Medical Literature:

"Proverbially, 'tis an ill wind that blows nobody good. The Eastern war illustrates this: The price of a human jaw at the seat of war in Bulgaria is, the *London Times* states, 10 *francs*, more or less. It varies according to the regularity, soundness, and whiteness of the teeth. In Paris, the quotation is fifty per cent. greater, at wholesale rates. The gastly wares are conveyed in cases containing five hundred, and the teeth are extracted after their arrival at the city to which the jaws are consigned."

*On the Elimination of Mercury.*—Dr. E. W. Hamburger, of Franzensbad, sums up a paper in the *Prager Medicin. Wochenschrift*, 1877, quoted in the London *Medical Record*, with the following conclusions:—

1. Mercury can be distinctly found in the urine of patients who have been treated with mercurial suppositories for some time. In one case, in which the treatment had been commenced four days previously, mercury was not found in the urine. Mercury is always present in the urine of patients who have been treated by inunction.

2. In patients treated with suppositories, mercury was always found in the milk as well as the urine. In cases of inunction, although mercury was present in the urine, none could be found in the milk; and, when mercurial inunction was substituted for suppositories, the mercury disappeared from the milk, although it continued to be present in the urine.

3. The fæces of a patient who was treated by inunction contained a large amount of mercury. Dr. Hamburger concludes, hence, that the elimination of mercury takes place chiefly by the bile.

The chemical process used consisted in the removal of organic matters, the application of electrolysis, and the formation of iodide of mercury, the crystals of which were readily recognizable under the microscope.—*Med. and Surg. Reporter.*

---

*Relation of Brain Weight to Mental Ability.*—Mr. C. Clapham says, in the last volume of the West Riding Lunatic Asylum Reports:—

"My observations agree with those of Wagner, that weight of brain does not indicate any close relation to intellectual power, and also that aboriginal races are not to be distinguished for smallness of brains. In fact, the ancient Britons, and I may add the ancient Gauls also, were remarkable for good-sized, nay, even large brains." This statement is borne out by the testimony of the most competent craniologists of the day.—*Med. and Surg. Reporter.*

---

*Curious Development of Teeth.*—Dr. L. E. Brayman, of Ohio, writes us:— My attention was called, the 30th of March, to a very peculiar malformation and unnatural development of the incisor teeth. Miss Alice G., of this place, has six incisors in the lower jaw, unnaturally flattened from side to side, and thick antero-posteriorly. In appearance they resemble the canine incisors, while on the upper jaw there are only two incisors, which are very broad, nearly filling the space between the canines. The jaws are somewhat flattened in front, but do not look very bad.—*Med. and Surg. Reporter.*

*Statistics of Insanity.*—In the Annual Report of the Pennsylvania Hospital for the Insane, for 1877, Dr. Kirkbride, the able superintendent, presents the statistics of 7663 cases admitted during the last 37 years. More than one-half (4248) were between the ages of 20 and 40. Of the three learned professions, 93 were physicians, 98 lawyers and 48 clergymen. As physicians outnumber lawyers more than two to one, and clergymen more than five to one, this showing is greatly in favor of the sanitary influence of a physician's life on the mind. Of the single females, 20 were daughters of physicians, 30 of lawyers, and 25 of clergymen—from which one might suspect that lawyers and clergymen, the latter particularly, transmit to their offspring a less healthy mental constitution. It is singular that a similar difference should appear among the wives. Of the married females, 25 were the wives of physicians, 49 of lawyers, and 32 of clergymen. Supposing the numerical ratio of the three professions in the community to be represented by the figures 1, 2 and 5, there would be 5 insane wives of physicians to 20 of lawyers and 32 of clergymen. Perhaps the hereditary tendency noticed among the daughters was on the mother's side. But why should the wives of doctors be more prone to insanity than those of the other professions? In the case of widows the proportion is somewhat reversed, 16 being widows of physicians, 16 of lawyers, and only 6 of clergymen.

---

*Dangers from Salicylic Acid.*—This is certainly a substance which should be administered with considerable caution. Several instances where it produced disagreeable consequences have been heretofore mentioned in these pages, and we note that Dr. Watelet communicates a paper to a late number of the *Bull. de Therap.* on this subject, entitled, "Accidents following the Administration of Salicylic Acid." The details are given of two cases of rheumatism treated by salicylate of soda, one of which was followed by gangrene of the lower extremities, and both by cystitis, obstinate constipation and coldness of the extremities.—*Med. and Surg. Reporter.*

---

*A Case of "Replantation of the Teeth."*—Ernst K., eight years old, while walking on stilts, fell down and knocked a central incisor entirey out, so that it had to be hunted for on the ground. After a long deliberation whether anything could be done with the tooth, the sister of the boy came with him to me for consultation.

The tooth was brought to me wrapped in a piece of paper; the roots were only developed to two-thirds of their full grown size and consequently the cavity of the pulp-chamber was still very large.

I cleaned the tooth carefully, by means of a syringe, with tepid water, cleaned the coagulated blood from the alveolus and pressed the tooth in as well as possible. In the absence of good gutta-percha, I heated some of Stent's composition and made a cap of it, covering the tooth in question and the adjacent ones, recommending forbearance of mechanic influences. During the following fortnight the cap was renewed three times and then dispensed with. Three weeks after the accident I found the tooth so solid in its socket that one would scarcely have believed that this tooth, three weeks before, had been about one hour out of the mouth.

It seems to me very remarkable in this case, that after the replantation, not a sign of inflammation or suppuration manifested itself, and that the tooth kept its color perfectly.—*Dental Office.*

---

*The Treatment of Earache.*—Dr. W. Cheatham, of Louisville, says, in a recent paper:

When a patient complains of earache, and on examination with the speculum the drum is seen to be red, it is good practice to turn into the ear a stream of water as warm as it can be borne. This is best done by the aural douche. Where this is not at hand, a Davidson's syringe may be substituted, first converting it, however, into a siphon. To do this, the vessel containing the water must be raised a short distance above the patient's head; the syringe then filled by compressing the bulb a few times, when, by lowering the tube, the water will continue to flow in a gentle stream, which is to be turned on the inflamed parts. A small rubber tube may be made to answer the same purpose. The douche, by whatever means affected, should be prolonged and often repeated.

Many cases of earache are met with, especially among children, which are relieved by having the patient turn the head well to the sound side, and pouring the ear full of very warm water. This may require to be repeated a number of times before relief is obtained, but in any event is always to be preferred to the various ear-drops, composed of laudanum, onion juice and the like. If this fails to relieve the pain, a leech should be applied a short distance inside the auditory canal, on its anterior wall; and when it falls away, the bleeding is to be encouraged by the hot water douche, or by flannels wrung from boiling water, industriously used for half an hour after. When the drum is found to be red and bulging, denoting fluid in the tympanic cavity, paracentesis should be immediately performed. The operation is exceedingly simple, and gives almost instantaneous relief. Should the fluid not flow as freely as may be desired, the patient is directed to practice Valsalva; or inflation should be made by Politzer's bag. In cases where the Eustachian tube is so entirely closed that air cannot be made to enter the middle ear, Seigel's otoscope, with very gentle suction, should be applied.—*Reporter.*

*Food and the Human Body.*—The basis of the science of food is in some degree uncertain. "No complete chemical examination of the total constituents of a healthy human body has yet been made," Prof. Church tells us, and the reason of this is obvious. We believe that in the course of the Franco-German war a number of soldiers killed in action were sent by the German authorities to an Italian laboratory. Whether the analyses were ever completed we do not know, but no results have been published. Meanwhile, we must be content with such approximations as have hitherto been made. It is probable that they are practically sufficient. The constituents of the human body, taking elements the names of which will be familiar to the general reader, rather than compounds, are 16 in number, seven of them being metals, nine non-metallic. The metals weigh altogether (11 stone, or 154 pounds, being taken as the standard weight of the whole body) something less than five pounds, nearly four of which are calcium, the basis of lime, supplying the chief part of the bones and teeth. Of iron there are 65 grains, a small amount, but very important, as giving color to the blood. Among non-metallic elements, oxygen is the most important, amounting to no less than 109 pounds: and next to this carbon, weighing not quite 19 pounds. Of phosphorus, which, if some physiologists are to be believed, supplies the motive power of the whole, there is 1 pound 12 ounces 25 grains. The weight of water in the body, to speak of compounds, not elements, is almost exactly the same as that of oxygen in the other list. The practical science of food is, of course, to keep up the supply of these substances to their normal quantity ; and here comes in the function of chemistry, which, having informed us what there is in the body, tells us what there is in each of the substances we commonly use to supply its needs.—*Druggists Circular.*

---

*Salicylic Acid as a Dentifrice.*—Herr Gehrke advocates its use in mouth-washes. Herr Zader held as an opposite experience that it is not to be so employed. Herr Kleinman infers from his experience that it acts injuriously on tooth bone, although but little upon the enamel. Herr Wiermann believes that the acid finds its way quickly through the fissures of the enamel.

The question was asked by Dr. Arend, whether observation had elicited the constant association of ill consequences in the mouth with the use of this acid.

Herr Buschendorf could vouch for such ill influences, as far as the necks of the teeth are concerned. Herr Elners had witnessed the association of pain as expressive of its irritating quality when applied to tooth bone and the dental neck. Herr Kleinman held it as a matter decided that the agent was corrosive to the teeth.—*Dental Cosmos.*

*Tubercular Ulcer of the Tongue.*—M. Nedopil remarks that the diagnosis of secondary tubercula ulcer of the tongue is generally not difficult, in the presence of other indications of tuberculosis. But primary tubercular ulcer can often be scarcely distinguished from cancer unless a microscopic examination be made, while the failure of anti-syphilitic remedies denotes that the affection is not a syphilitic ulcer, which often has a similar appearance. The tubercular ulcer of the tongue runs a course resembling that of cancer. A small hard nodule on the edge or upper surface of the tongue, which is often overlooked, at last falls off and leaves a dirty ulcer with an indurated base, which generally spreads more slowly than a cancerous ulcer. Early extirpation is the only cure, and it may perhaps arrest the development of general tuberculosis. The author has observed four cases in Billroth's clinic; two of the individuals were thirty-two years of age, the others sixty-eight and seventy. In three cases the ulcer was extirpated, and healing took place in a few days. In the excised pieces the tissue around the ulcer was studded with miliary-tubercules, mostly towards the free surface. The morbid process appears to commence with a general transformation of the muscular tissue into a homogenous, slightly granular plasma containing proliferating muscle-nuclei. Later, the primary deposits become confluent, and giant-cells are formed from the obstructed portions of the blood-vessels; in some of these, Nedopil found cavities filled with brown pigment. The growth of the tubercle appears to take place partly through proliferation of nuclei (without cell-formation) in the interior, partly through metamorphosis of the neighboring tissue.—*British Medical Journal.*

---

*The Cell and Protoplasm.*—In a lecture recently delivered at the Royal Institution, Professor A. H. Garrod said that he believed the original idea of a cell, as first taught by Schleiden and Schwan, is incorrect. The use of the reagents they employed, to get clearness as they supposed, really brought about artificially those changes which led them to believe that a cell consisted of cell wall, cell contents, nucleus, and nucleolus. He would define as a cell a separate mass of protoplasm, whether surrounded by formed material or not. This formed material comes from the precipitation of salts of lime by the protoplasm and from the formation of hyaline, etc. In this way the tissues of the body are built up. In the growth of the epidermis, the cells are gradually more and more filled with precipitated matter, the protoplasm occupies less and less space, and finally the cells die and are removed from the surface. In fatty tissue, the hydrocarbons of the food are gradually precipitated in the cells till the protoplasm becomes only an investing membrane.—*Med. and Surg. Reporter.*

